# Manners

**Published:** 1873-11

**Authors:** 


**Manners.**—I wish cities would teach their best lesson, of quiet manners. It is the foible, especially of American youth, —pretension. The mark of the man of the world is absence of pretension. He does not make a speech; he talks in a low business tone, avoids all brag, is nobody, dresses plainly, promises not at all, performs much, speaks in monosyllables, hugs his fact. He calls his employment by the lowest name, and so takes from evil tongues their sharpest weapon. His conversation clings to the weather and the news, yet he allows himself to be surprised into thought, and the unlocking of his learning and philosophy. How the imagination is piqued by anecdotes of some great man passing incognito, as a king in gray clothes! of Napoleon affecting a plain suit at his glittering levée! of Burns, or Scott, or Beethoven, or Wellington, or Goethe, or any container of transcendent power, passing for nobody! of Epaminondas, "who never says any thing, but will listen eternally!" of Goethe, who preferred trifling subjects and common expressions in intercourse with strangers, worse rather than better clothes, and to appear a little more capricious than he was! There are advantages in the old hat and box-coat. I have heard that throughout this country, a certain respect is paid to good broadcloth; but dress makes a little restraint; men will not commit themselves. But the box-coat is like wine; it unlocks the tongue, and men say what they think.—RALPH WALDO EMERSON.

Another writer says: The young should be mannerly, but they feel timid, bashful and self-distrustful the moment they are addressed by a stranger or appear in company. There is but one way to get over this feeling, and acquire easy and graceful manners, and that is, to do the best they can at home as well as abroad. Good manners are not learned so much as acquired by habit. They grow upon us by use. We must be courteous, agreeable, civil, kind, gentlemanly, and manly at home, and then it will become a kind of second nature every where. A coarse, rough manner at home begets a habit of roughness, which we can not lay off if we try, when we go among strangers. The most agreeable persons in company are those who are most agreeable at home. Home is the school for all the best things.

